# Three-year follow-up of antibiotics versus surgery for acute appendicitis

**DOI:** 10.1308/rcsann.2026.0018

**Published:** 2026-02-23

**Authors:** S Bose, HJ Wong, C Skouras, Y Maeda

**Affiliations:** ^1^ Queen Elizabeth University Hospital, UK; ^2^ University of Glasgow, UK

**Keywords:** Appendicitis, Appendectomy, Recurrence, Antibiotics

## Abstract

**Introduction:**

Acute appendicitis is one of the most common surgical emergencies. Emerging evidence suggests uncomplicated appendicitis could be treated by antibiotics. We investigated the outcome of patients treated with antibiotics for acute appendicitis with a three-year follow-up. Secondary aims were to assess recurrence of appendicitis and potential missed pathologies with nonoperative management.

**Methods:**

All computed tomography (CT) scan reports with keyword ‘appendicitis’ of adult patients performed from January 2020 to December 2021 were reviewed. Reports were classified as uncomplicated or complicated based on previous definition. Demographic data, treatment information including operative details and complications, and recurrence of antibiotic-treated appendicitis were collected retrospectively.

**Results:**

Among 3,252 scans, 1,008 confirmed appendicitis (631 uncomplicated, 377 complicated; median age 46 years). 212 patients (21.0%) were treated with antibiotics and 796 (79.0%) underwent appendicectomy. After excluding deaths, 195 nonoperatively treated patients were available for three-year follow-up: 83 (42.6%) developed recurrence and 57 (29.2%) had surgery. Neoplasms were found in 19 of 591 (3.2%) CT-diagnosed uncomplicated appendicitis. Overall, neoplasms were found in 3.5% of the specimens. Postoperative complications occurred in 11% of all surgeries, of which 2.3% were Clavien–Dindo grade III–V.

**Conclusions:**

Antibiotic treatment of appendicitis is associated with high recurrence and risk of missed neoplasms. Appendicectomy is safe, with low rates of significant complication. When offering nonoperative management for appendicitis, patients should be informed about the risk of recurrence and missing sinister pathologies.

## Introduction

Acute appendicitis is one of the most common surgical emergencies worldwide, yet its optimal management remains the subject of active debate. The traditional approach of prompt appendicectomy has been challenged in recent years by emerging evidence supporting nonoperative management in selected cases. Recent randomised controlled trials (RCTs) have evaluated the effectiveness of antibiotics in the management of appendicitis. Most of these trials had a median follow-up of approximately one year, reporting recurrence or need for surgery in 25–41% of patients.^1–5^ Importantly, these figures excluded cases with early treatment failure following antibiotic therapy, suggesting that the true recurrence rate is likely higher.

In parallel, recent demographic reports have highlighted a steady rise in the incidence of appendiceal malignancy, particularly neuroendocrine tumours (NETs), with the increase being more pronounced among younger patients. An English population-based study showed that the incidence of appendiceal cancer rose from 0.4% in 1999 to 2% in 2016.^6^ In the United States, one report documented a 54% increase in appendiceal cancer incidence between 2000 and 2009,^7^ while a more recent North American study (US and Canada) found a more than twofold rise in incidence between 2000 and 2015.^8^

The long-term outcomes of acute appendicitis managed by antibiotics and the incidence of missed pathology remain scarcely researched. The primary aim of this study was to assess antibiotic management of uncomplicated appendicitis. Secondary aims were to evaluate recurrence of appendicitis during a three-year period and incidence of malignancy.

## Methods

### Study design and selection

Consecutive computed tomography (CT) scan reports from January 2020 to December 2021 at NHS Greater Glasgow and Clyde (NHS GGC) were reviewed. NHS GGC is the largest NHS board in the UK, serving a population of approximately 1.3 million. All patients who underwent CT imaging with clinical suspicion of acute appendicitis were identified using the search keyword ‘appendicitis’, both in request and conclusion to allow permissive search. The Picture Archiving and Communication System (PACS) of NHS GGC is the platform on which this keyword search was performed. Each scan report was then reviewed to determine eligibility for this study by two persons (SB and HJW).

Patients were included if they were 16 years of age or older, had a CT-confirmed diagnosis of acute appendicitis and required hospital admission. To ensure comparability between treatment groups, we excluded patients not fit for surgical treatment. Patients were also excluded if the CT scan showed no evidence of appendicitis, or if they had a previous documented history of appendicitis on electronic patient records (i.e., not index episode of appendicitis). Further exclusions applied to duplicate imaging studies, cases with missing treatment records and CT scans performed for cancer staging rather than for suspected appendicitis.

Data for all eligible patients were collected from electronic medical records (Trakcare and Clinical Portal).

### Classification of complicated and uncomplicated cases

Appendicitis described by the terms ‘uncomplicated appendicitis’, ‘simple appendicitis’, ‘early appendicitis’ or ‘appendicitis without perforation’ on CT reports and not describing any feature of complicated appendicitis were classified as uncomplicated. Appendicitis was classified as complicated if the CT demonstrated any high-risk features, as previously described in literature.^9,10^ These high-risk features include extraluminal free air, peri-appendiceal fluid, presence of an appendicolith, moderate-to-severe peri-appendiceal fat-stranding and a visible defect in the appendiceal wall and/or ileus.

### Definition of treatment

Patients were classified into two groups:

1. The antibiotic treatment group—those who received at least 12 hours of intravenous or oral antibiotics and did not undergo appendicectomy during the hospital admission associated with the CT scan.
2. The appendicectomy group—those who underwent surgery to remove the appendix during the same hospital admission. Postoperative complication and histology results for the patients in the second group undergoing surgery was also recorded.

Statistical analyses were performed using R (version 4.5.1). Normality of data distribution was assessed using the Shapiro–Wilk test. For nonparametric data, comparisons between two groups were carried out using the Mann–Whitney *U* test. Categorical variables were analysed using the Chi-square test or Fisher’s exact test, as appropriate. A *p*-value<0.05 was considered statistically significant.

Access to, and use of, data collected during routine clinical care was reviewed and approved by the Caldicott Guardian.

## Results

Between January 2020 and December 2021, a total of 3,252 CT scans contained the keyword ‘appendicitis’. Of these, 2,244 scans were excluded according to the criteria; the numbers and reasons of exclusions are listed in Figure 1. The remaining 1,008 cases were included in the study (Figure 1). Based on the predefined criteria, 631 patients (62.6%) were classified as having uncomplicated appendicitis, while 377 patients (37.4%) were classified as having complicated appendicitis.

**Figure 1:**
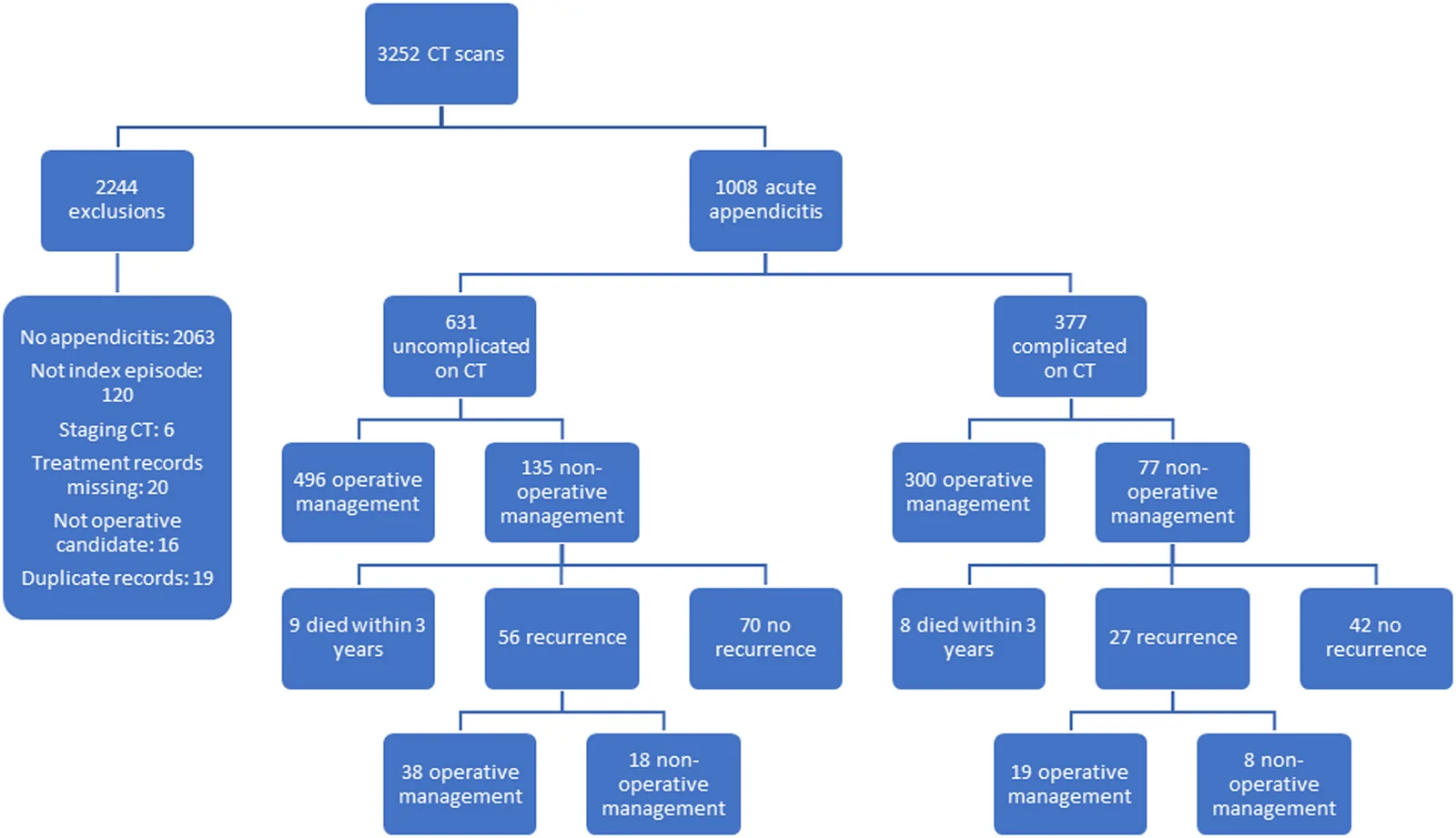
Prisma flow diagram for patient inclusion and exclusion CT = computed tomography

The cohort consisted of 1,008 patients, including 544 male patients, with a median age of 46 years (range 16–98 years). Among these patients, 51 (5%) had diabetes and 13 (1%) were receiving long-term oral steroid therapy.

All patients had C-reactive protein measured at the time of admission, with values ranging from 1 to 569mg/L and a median of 84mg/L. Body mass index was documented in 42% of cases, with values ranging from 16 to 50kg/m² and a median of 28kg/m².

The median length of hospital stay was 3 days (range 0–66 days). Among patients who underwent surgery, the American Society of Anesthesiologists physical status classification is summarised in Table 1.

**Table 1:** Summary of demographic data, radiological findings and operative information

		CT Uncomplicated	CT Complicated
Age	Median	45	48
Sex	Male	334	210
	Female	297	167
Hospital stay (days)	Median	3	5
ASA	1	186	83
	2	223	163
	3	34	33
	4	2	1
	5	0	0
	Not documented	51	20
Diabetes	No	602	355
	Yes	29	22
CRP	Median	63	140
Treatment groups	Nonoperative	135	77
	Surgery	496	300
Approach	Laparoscopic	417	189
	Open	38	29
	Laparoscopic converted to open	39	64
	Laparotomy	2	18
Operative finding	Uncomplicated	381	78
	Complicated	112	222
	Normal	3	0
Histology (index)	Appendicitis	480	279
	Cancer	8	15
	IBD	1	3
	Normal	4	0
	Other	1	3
	Appendicectomy not done	2	0
Recurrence	Yes	56	27
	No	70	42
Treatment of recurrent appendicitis	Laparoscopic	32	11
	Open	3	3
	Laparoscopic converted to open	2	1
	Laparotomy	1	4
	Nonoperative	18	8

ASA = American Society of Anesthesiologists; CRP = C-reactive protein; CT = computed tomography; IBD = inflammatory bowel disease

### Treatment path

Of 1,008 patients with acute appendicitis, 212 (21%) were treated nonoperatively. In this group, there was one patient who had antibiotics and percutaneous drainage of abscess. The remaining 796 patients (79%) were treated surgically, as summarised in Figure 2.

**Figure 2:**
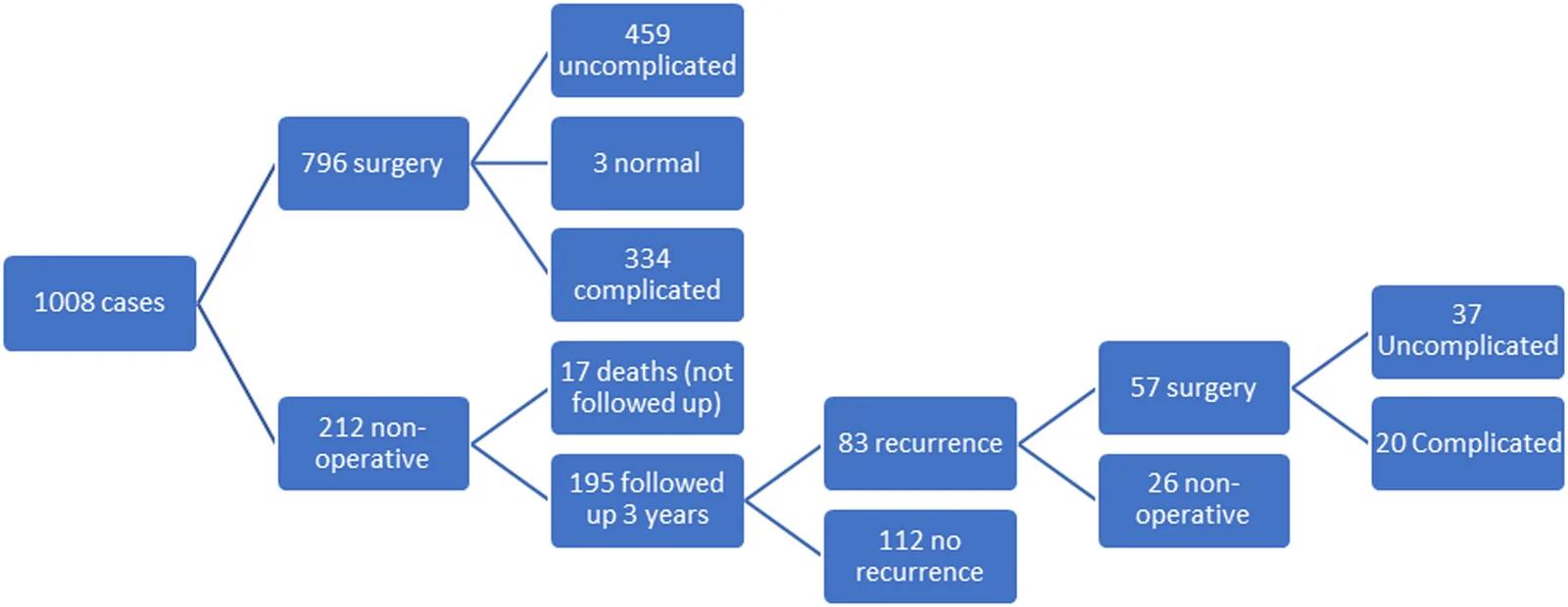
Final distribution of patients based on treatment pathway

Surgical approaches were laparoscopic in 606 (76.1%) patients, laparoscopic converted to open in 103 (12.9%) patients, open in 67 (8.4%) patients and laparotomy in 20 (2.5%) patients. Operative findings were uncomplicated appendicitis in 459 (57.7%) patients, complicated appendicitis in 334 (42.0%) patients and macroscopically normal in 3 (0.4%) patients. Time from the diagnostic scan to operative induction was a median of 665.5 minutes (range 27 minutes to 187.2 hours).

### Recurrence of appendicitis

Among the 212 patients initially treated with antibiotics, 17 (8.0%) died within three years of their index episode of appendicitis with causes unrelated to appendicitis, leaving 195 patients available for recurrence assessment. Among these, appendicitis recurred in 83 patients (42.6%). For treatment of recurrence of appendicitis 57 of 83 patients (69%) underwent surgery. Histological analysis confirmed acute appendicitis in 50 patients (88%), while 7 patients (12%) were found to have appendiceal neoplasms.

A detailed breakdown of all the cases including recurrence based on CT finding of complicated or uncomplicated appendicitis is depicted in flowchart form in Figure 1.

Of 212 patients who underwent antibiotic treatment, 135 (63.7%) had uncomplicated appendicitis while 77 (36.3%) had complicated appendicitis on CT. Excluding patients who died of causes unrelated to appendicitis, 126 patients in the uncomplicated group and 69 patients in the complicated group were available for outcome review at three years. The recurrence rates were 56 of 126 (44.4%) patients in the uncomplicated group and 27 of 69 (39.1%) patients in the complicated group. There was no statistically significant difference between the two groups (*p*=0.47). The categorisation of the 195 patients available for recurrence assessment is depicted in graphical form in Figure 3.

**Figure 3:**
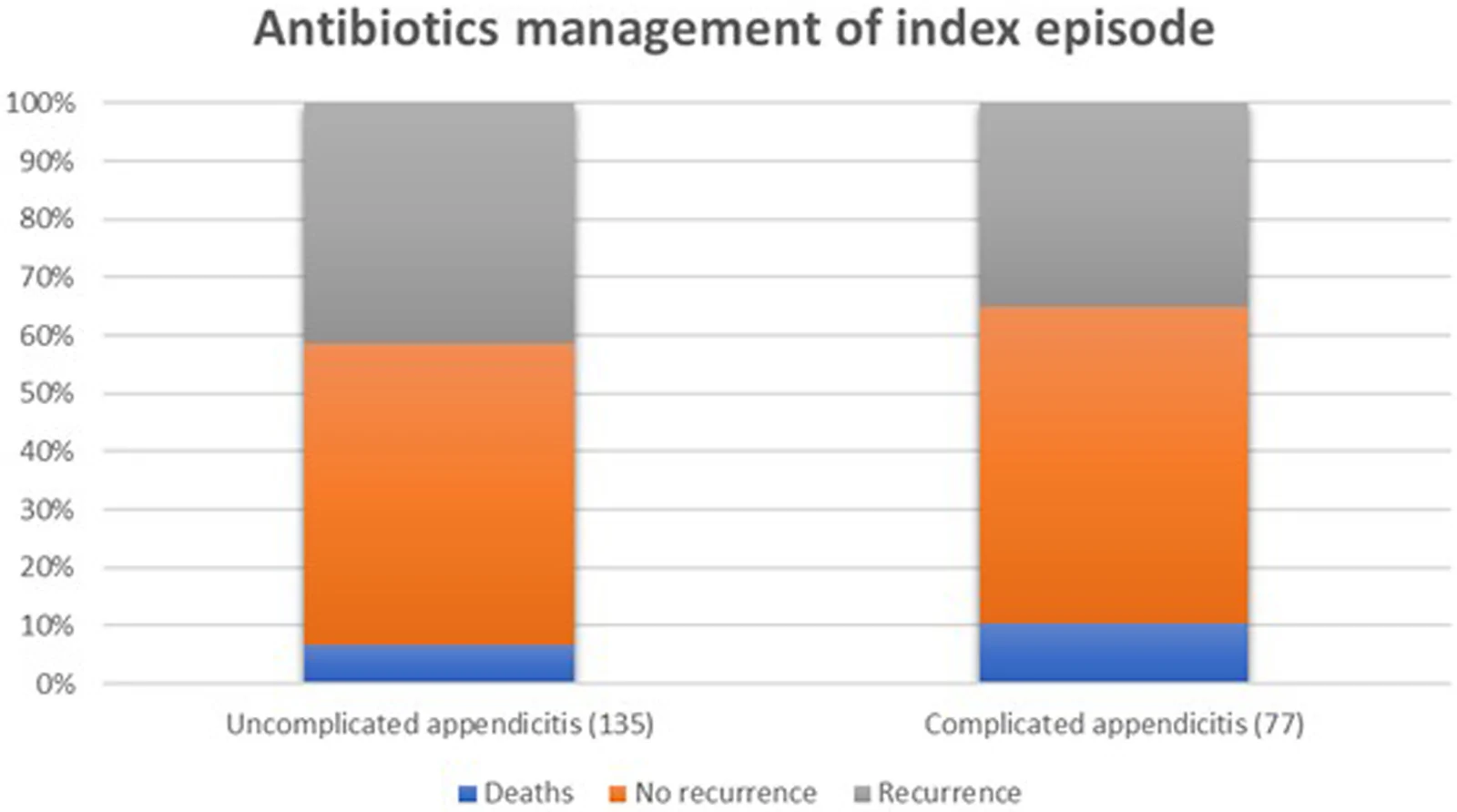
Outcome of appendicitis when managed with antibiotics only

### Operative findings of recurrent cases

Among the recurrent cases, 38 patients with an initial uncomplicated CT diagnosis underwent surgery, with intraoperative findings confirming uncomplicated disease in 31 and complicated disease in 7. Conversely, 19 patients with a complicated CT diagnosis underwent surgery, of whom 13 had complicated disease, while 6 were found to have uncomplicated appendicitis intraoperatively.

### Complications

Of 796 patients undergoing surgery during index admission, 88 (11%) had a complication; the majority (77%; 68 of 88) were Clavien–Dindo (CD) grade I or II complications,^11^ 18 (21%) had CD grade III or IV and there were 2 (2%) deaths (CD grade V) after surgery. The overall 30-day mortality was four (0.4%) in the entire study population, with two of the deaths being in the nonoperatively managed cohort. Of 57 patients who had surgery due to recurrent episodes, 2 (4%) had postoperative complications (CD grades I and II only).

### Histology

Histology including both index and recurrent episodes (total 853) showed appendicitis in 809 (94.8%) patients. The analysed specimens of 30 (3.5%) patients showed neoplasms, 4 (0.5%) patients had inflammatory bowel disease, 4 (0.5%) had normal appendix, 4 (0.5%) patients had other pathologies (2 appendiceal polyp, 1 neurogenic appendicopathy and one specimen contained fat only and no appendix), and 2 (0.2%) patients who had a diagnostic laparoscopy but not appendicectomy because their appendix looked normal intraoperatively.

Adenocarcinoma was the most common malignancy encountered (13 of 30) and Goblet cell adenocarcinoma the second most common (7 of 30). There were four T3 or T4 low-grade appendiceal mucinous neoplasms and three NETs of appendix. The other three were rarer tumours: gastrointestinal stromal tumour, serous carcinoma and chronic lymphocytic leukaemia.

## Discussion

Currently, there is no robust evidence supporting the long-term effectiveness of nonoperative management of acute appendicitis. Most published studies have focused primarily on immediate outcomes, such as short-term symptom resolution, rather than long-term recurrence rates or potential adverse consequences.^1,5,12–14^ In this study, we aimed to evaluate not only immediate outcomes but also medium-term recurrence and subsequent management. We included only those who could be considered for both of the treatment options. This approach allowed us to better capture the reality of daily clinical practice of patients who could be treated by either surgical or nonoperative management of acute appendicitis.

Our recurrence rate of 42.6% is alarmingly high and strongly suggests that nonoperative management may not be the most effective option for the majority of patients. By contrast, the rate of significant postoperative complications (CD grade III–V) following appendicectomy was low at 2.3% (20 of 853). Longer-term data from key trials raise concerns as well. The APPAC trial reported a 39% recurrence rate at five years,^15^ while another study with a seven-year follow-up demonstrated a similar 39% recurrence rate in patients managed with antibiotics.^2^ Notably, the latter also reported poor patient satisfaction among those experiencing recurrence, with no significant difference in satisfaction between patients treated with surgery versus antibiotics.^2^ Furthermore, the COMMA trial demonstrated inferior quality-of-life outcomes in the antibiotic group compared with surgically treated patients.^1^ These data emphasise the importance of discussing recurrence risk with patients when considering nonoperative management.

The risk of recurrence holds equally true for both uncomplicated and complicated cases as shown in our results; there was no statistically significant difference between the odds of recurrence between uncomplicated and complicated cases. This is in contrast to the customary view that complicated appendicitis recurs more often compared with uncomplicated. To stress this further, the presence of appendicolith has been found to cause early treatment failure and complications rather than future recurrence.^3^ We have not emphasised the different outcomes from the presence of appendicolith in our study; this has been included in the complicated group as defined in Methods and previous literature. As our study design is retrospective, it was not possible to ascertain how many patients had early treatment failure rather than delay in surgery, and whether the presence of appendicolith had any impact on treatment failure. Radiology reports do not always specify the presence of appendicolith, and the absence of documentation in the report is not equivalent to absence of appendicolith. On balance, we felt that retrospective review of all imaging was out of the scope of the aim of this study and this specific point was not investigated. Further research on this particular point will be useful to evaluate in detail in a prospective study with accurate reporting of presence or absence of appendicolith and a clear definition of treatment failure.

An additional concern is the diagnosis of neoplasms. In our cohort, tumour lesions were detected in 3.5% (30 of 853) of cases, underscoring the risk of missing underlying neoplasia when appendicitis is managed nonoperatively. This finding aligns with recent literature highlighting an increasing incidence of appendiceal cancer.^6–8^ Furthermore, when looking at tumour occurrence in the recurrence group, this was alarmingly high at 12.3% in our study. This raises a possibility of missing the underlying malignancy when the lesion is flat and thus may be less obvious on CT scan. The overall tumour rate in nonoperatively managed appendicitis within three years was 3.3% (7 of 212), suggesting a similar rate throughout the study population of 1,008, irrespective of their initial management pathway (surgical vs nonoperative).

A key limitation of this study is the lack of detailed insight into the decision-making process for nonoperative management. Consequently, it remains unclear whether nonoperative treatment was primarily the result of patient preference, clinician recommendation or the influence of comorbidities that made surgery less favourable. This restricts our ability to determine whether nonoperative management reflected patient-centered choice or medical necessity. Although a prospective set-up to evaluate this will provide more information on this specific subject, there will always be a concern of a degree of bias when the patients are involved in ‘research’. Furthermore, comprehensive comorbidity data were not collected, and validated risk assessment tools such as NELA or P-POSSUM were not applied, limiting the objective evaluation of surgical fitness across the cohort. Finally, patient perspectives were not captured, including satisfaction with treatment, perceptions of long-term outcomes and the rationale underpinning management choices. These factors represent important gaps that future studies should address to better inform both clinical decision-making and patient experience in the management of appendicitis.

The other limitation to consider is that the study period falls within the COVID-19 pandemic timeline. We do acknowledge that this might have some effect on the decision making towards a surgical or antibiotics approach to manage appendicitis, but in no circumstance would this affect the risk of recurrence or underlying malignancy, which was our main focus to unravel. We have noticed a higher number of open surgeries were carried out compared with standard laparoscopic surgery in that time period, and this may actually increase the number of complications in our study. It is practically impossible to quantify how many of these decisions were made due to patient factors and how many solely due to COVID-19.

## Conclusion

In this large cohort study (*n*=1,008), nonoperative management with antibiotics showed a high recurrence rate of 42.6%.The risks associated with nonoperative management outweigh its potential benefits. Serious surgical complications categorised as CD III, IV or V occurred in only 2.3% of cases, while the risk of missing an underlying neoplasm was higher at 3.5%. Nonoperative treatment carries a high risk of recurrence, fails to address the possibility of underlying tumour and offers no clear long-term advantage over surgery.

## Competing interests

All the authors declare there are no conflict of interest.

## Funding

The author/s received no financial support for the research, authorship and/or publication of this article.

## Ethical clearance

Appropriate ethical approval was obtained from the ethical committee of the institution where this study was performed.

## Author contributions

**S Bose:** Conceptualisation, Data curation, Formal analysis, Investigation, Methodology, Project administration, Software, Supervision, Validation, Visualisation, Writing – original draft, Writing – review & editing. **HJ Wong:** Data curation, Resources. **C Skouras:** Conceptualisation, Visualisation, Writing – review & editing. **Y Maeda:** Conceptualisation, Data curation, Formal analysis, Methodology, Project administration, Software, Supervision, Validation, Writing – original draft, Writing – review & editing.

## Artificial Intelligence

The author/s declare that no AI was used to conduct the study or prepare the manuscript.
